# 

**Published:** 1860-07

**Authors:** 


### CONTENTS FOR JULY, 1860.

### ORIGINAL COMMUNICATIONS.

Medical Teachers' Association, ........ 385
Thirteenth Annual Meeting of the American Medical Association, ........ 392
Surgical Notes. By E. ANDREWS, M. D., ........ 413
Correspondence of Prof. W. H. Byford, ........ 415
Clinical Reports. By Prof. N. S. DAVIS. Mercy Hospital, ........ 420
Prof. DAVIS' Clinique in the Medical Department of Lind University, ........ 428

### BOOK AND PAMPHLET NOTICES.

Transactions of the Medical Sooiety of the State of New York, ........ 431
Second Anuual Report of the Chicago Charitable Eye and Ear Infirmary, ........ 432
Forty-Third Annual Report on the State of the Asylum for the relief of persons deprived of the use of their reason, ........ 433
Proceedings of the Sixty-Eighth Annual Convention of the Connecticut Med. Society, .. 433

### SELECTIONS.

Parisian Medical Intelligence ........ 434
Relation of the Medical Profession to the Public, ........ 437
Trial for Abortion, ........ 437
A Bribe, ........ 438
Longevity, ........ 438
Man-Eating Tigers ........ 438
Influence of Slow-Lead Poisoning on the Product of Conception ........ 438
The Dietetic Inventiveness of Hunger ........ 439
The Sphygmographer, or Register of the Arterial Pulse, ........ 440
Rasorian Doses of Tartar Emetic in Tetanus, ........ 440
Drowning in a Water Bed, ........ 440
Dissecting Wounds, ........ 440
A New Military Hospital in Paris, ........ 441
Cephalotripsy without Traction versus the Caesarean Operation ........ 441
Mortality in the Russian Capitols ........ 441
Medicines of the Shetlanders, ........ 441

### EDITORIAL.

American Medical Association, ........ 442
Convention of Delegates from Medical Colleges ........ 442
Medical Colleges, 1859-60 ........ 444
Notice to Medical Men in Illinois ........ 445
To the Medical Profession of Illinois ........ 445
Hydrocynate of Iron in Epilepsy ........ 446
Medicine in China ........ 446
Malformation of the Chest ........ 447

